# Effects of the *Aphanizomenon flos-aquae* Extract (Klamin®) on a Neurodegeneration Cellular Model

**DOI:** 10.1155/2018/9089016

**Published:** 2018-09-17

**Authors:** D. Nuzzo, G. Presti, P. Picone, G. Galizzi, E. Gulotta, S. Giuliano, C. Mannino, V. Gambino, S. Scoglio, M. Di Carlo

**Affiliations:** ^1^Istituto di Biomedicina ed Immunologia Molecolare (IBIM) “Alberto Monroy”, CNR, Via Ugo La Malfa 153, 90146 Palermo, Italy; ^2^Chemical Laboratory of Palermo, Italian Agency of Customs and Monopolies, Via Crispi, 143, 90133 Palermo, Italy; ^3^Nutritherapy Research Center, 61029 Urbino, Italy

## Abstract

Cyanobacteria have been recognized as a source of bioactive molecules to be employed in nutraceuticals, pharmaceuticals, and functional foods. An extract of *Aphanizomenon flos-aquae* (AFA), commercialized as Klamin®, was subjected to chemical analysis to determine its compounds. The AFA extract Klamin® resulted to be nontoxic, also at high doses, when administered onto LAN5 neuronal cells. Its scavenging properties against ROS generation were evaluated by using DCFH-DA assay, and its mitochondrial protective role was determined by JC-1 and MitoSOX assays. Klamin® exerts a protective role against beta amyloid- (A*β-*) induced toxicity and against oxidative stress. Anti-inflammatory properties were demonstrated by NF*β*B nuclear localization and activation of IL-6 and IL-1*β* inflammatory cytokines through ELISA. Finally, by using thioflavin T (ThT) and fluorimetric measures, we found that Klamin® interferes with A*β* aggregation kinetics, supporting the formation of smaller and nontoxic structures compared to toxic A*β* aggregates alone. Altogether, these data indicate that the AFA extract may play a protective role against mechanisms leading to neurodegeneration.

## 1. Introduction

Blue-green algae, including *Aphanizomenon flos-aquae* (AFA), are unicellular prokaryotic microorganism belonging to the Cyanobacteria (Cyanophyta) phylum. These bacteria are among the oldest life forms, are the only microorganisms to achieve oxygenic photosynthesis, and probably have been the main biotic source of oxygen on early Earth [1]. These microorganisms are widely diffused, colonizing both salt- and freshwater around the world, alone or in a symbiotic relationship with plants or fungi [2]. Cyanobacteria are a source of bioactive compounds such as polyunsaturated fatty acids, proteins, pigments, and minerals and are rich in substances with anti-inflammatory and antioxidant properties [3–8].

The Aphanizomenon genus includes several species including the flos-aquae. AFA is a cyanobacterial unicellular organism endowed with several health-enhancing properties that, unlike other commercial “microalgae,” spontaneously grows in Upper Klamath Lake (southern Oregon, USA) where it also inhibits, when in bloom, the growth of other cyanobacterial species. Upper Klamath Lake is an ideal natural ecosystem, in which the AFA microalga finds the perfect conditions that allow its proliferation, especially between late summer and early fall, while going quiescent in the winter. The 300 days of sunshine that characterize the Klamath Basin favor its intense photosynthetic activity, managed by the microalga through various pigments, including its unique type of phycocyanins. Being placed at 1300 meters of height, in the winter, the lake freezes over, a condition which stimulates the production of fatty acids, including omega 3, by the AFA microalgae. AFA contains a high concentration of vitamins, especially of the B group. Vitamin B12 is essential for the synthesis of nucleic acids, of erythrocytes, and for myelin formation. B12 deficiency can cause a series of more or less severe nervous system-associated symptoms [9, 10]. Due to the volcanic origin of the lake, AFA contains a wide and complete spectrum of minerals and trace minerals [11]. It is also rich in pigments such as carotene, beta-carotene, and chlorophylls [12]. Of particular relevance are its phycocyanins, which have a particular structure [13] and have proven to have significant antioxidant [14], anti-inflammatory [15], and antiproliferative [16] properties.

Furthermore, AFA contains significant amounts of phenylethylamine (PEA), an endogenous molecule that is considered a general neuromodulator [17] and which is lacking in certain forms of depression and affective disturbances [18, 19]. Klamin® is an AFA extract which concentrates phenylethylamine, as well as other molecules, such as AFA-phycocyanins and the mycosporine-like amino acids (MAAs) porphyra and shinorine, which act as powerful MAO-B inhibitors [20]. Klamin® has proven to be effective in countering depression, anxiety, and other pathologies [21–23]. This is why Klamin® is emerging as a nutritional supplement supporting the correct functioning of the neurological system, and its role in neurodegenerative diseases has been reported [24]. The PEA contained in Klamin® has also been shown to play a role in modulating the immune system response [25]. All these properties make Klamin® a potential therapeutic agent, especially for those pathologies in which oxidative stress, inflammation, and mitochondrial and neurological dysfunction play a relevant role.

With the increase of the life expectancy, the number of people affected by neurodegenerative diseases is growing. Among these diseases, Alzheimer's disease (AD) is the most diffuse form of dementia [26–29]. Currently, no effective treatment is available. Hence, there is a great interest in studying natural bioactive compounds to use as neuroprotective and neuroregenerative agents. One of the histopathological hallmarks of AD is the amyloid plaque formation in the brain. These plaques are mainly constituted by aggregates of the A*β* peptide, a peptide generated by proteolytic cleavage of the amyloid precursor protein (APP) [30]. At the molecular level, A*β* induces oxidative stress, inflammation, mitochondrial dysfunction associated with specific signaling impairment, and apoptosis [31–36]. Antioxidant molecules, such as ferulic acid, have been successfully employed to inhibit oxidative stress, mitochondrial dysfunction, and apoptosis in an *in vitro* model of Alzheimer's [37–39]. An attractive approach for preventing or reducing neurodegeneration could consist in introducing dietary supplements with antioxidant, anti-inflammatory, and neuromodulating properties. Antioxidant and anti-inflammatory substances from natural, food-derived sources display elevated bioavailability and a higher efficacy than synthetic antioxidants that do produce scarce if any effect [40]. Thus, a proper intake of natural, food-derived antioxidant and anti-inflammatory substances can play an important role in preventing and controlling diseases. Some studies have shown that the AFA extract Klamin® can improve mood, reduce anxiety, and enhance attention and learning, suggesting that it could have a role in clinical areas including mood disorders and neurodegenerative diseases [20, 21].

In the present study, we investigate some biological properties of Klamin®'s compounds, thus evaluating the possibility to use it as a dietary supplement playing a protective role against neurodegenerative disease. A soluble preparation of the AFA extract Klamin® was used to identify the antioxidant properties in a neuronal LAN5 cell model and its protective effect on neurodegeneration by biochemical assay and inhibition of A*β* fibrillogenesis kinetics.

## 2. Materials and Methods

### 2.1. Solubilization of Klamin®

The Klamin® extract was kindly provided by Nutrigea Research s.r.l. (Republic of San Marino). Klamin® tablets were pulverized using a mortar and pestle and 10 mg of powder was dissolved in 10 ml of PBS (pH = 7.4; 137 mM NaCl, 2.7 mM KCl, 8 mM Na_3_PO_4_). The solution was sonicated (70% of the maximum power, twice for 30 seconds) and magnetically stirred for an hour. The insoluble faction was removed by centrifugation at 14,000 rpm for 30 min at 4°C. The supernatant was collected, filtered by using a 0.45 *μ*m Sartorius filter, aliquoted (1 ml/vial), and stored at −20°C. We will call this fraction “AFA extract.”

### 2.2. Chemical Analysis

The solubilized Klamin® (1 : 100) was loaded on a cuvette and measured by using a spectrophotometer in the range between 310 and 360 nm for mycosporine-like amino acid (MAA) identification and in the spectrum range of 400–600 nm for carotenoid, phycoerythrin, and phycocyanin identification. Phyco-complexes were visualized by using a fluorescence microscope (ZEISS) and specific excitation filters for UV (blue emission), FITC (green emission), and Texas Red (red emission). Phenolic compounds were determined according to Ignat et al. [41] and Naczk and Shahidi [42] and by employing reverse-phase high-performance liquid chromatography (HPLC) combined with a photodiode array detector (DAD) (Accela Thermo Fisher Scientific) and UV detection at 280 nm wavelength (Zang et al., 2013) and by using 3-hydroxytyrosol (Sigma), caffeic acid (Fluka), and vanillic acid (Fluka) as standard. The column was a C18 THERMO Accucore (2.1 mm × 100 mm, particle size 2.6 *μ*m) thermostated at 30°C. The mobile phase was obtained by a gradients composed by 50% methanol/50% acetonitrile (solution A) and H_2_O/0.2% H_3_PO_4_ (solution B). The flow rate is 450 *μ*l/min. The identity of the phenolic compounds was ascertained by comparing their retention times and UV–Vis spectra with that of authentic standards. Carbohydrates were analyzed according to Rojas-Escudero et al. [43] and by using a gas chromatography-mass spectrometry (GC-MS) (GC-MS ISQ LT 300 Thermo Fisher Scientific) instrument and D-Pinitol (Sigma) and inositol (Sigma) as standard. The solubilized Klamin® sample (1 ml) was submitted to the derivatization method to increase its volatility by using SYLON HTP kit HDMS+TMCS+PYRIDINE 3 : 1 : 9 Supelco at 80°C for 1 hour. Then, the sample was centrifuged at 5000 rpm for 30 minutes and the supernatant was freeze-dried in nitrogen. After the addition of hexane (1 ml), the sample was analyzed by GC-MS. Mineral compounds were evaluated by inductively coupled plasma-mass spectrometry (ICP-MS) (ICP X-SERIES 2 Thermo Scientific Instrument). The AFA extract (5 gr) was ionized by microwave digestion by using a MARS Xpress instrument (CEM) and in the presence of nitric acid (10 ml) at 400 W for 15 minutes and at 800 W for additional 15 minutes. After digestion, 500 ml of H_2_O was added to the sample for qualitative and quantitative mineral determinations by mass spectrometry.

### 2.3. Folin-Ciocalteu Colorimetric Assay

Hydrosoluble biophenolic content was determined by using Folin-Ciocalteu colorimetric assay.

An aliquot (0.2 ml) of the AFA hydrosoluble extract was diluted with distilled water to a total volume of 5 ml, and 0.5 ml of the Folin-Ciocalteu reagent was added. After 3 min, 1 ml of Na_2_CO_3_ (20% *w*/*v*) was added to the reaction mixture that was mixed and diluted with water to 10 ml. The samples were stored for 2 hours at room temperature, and the absorbance of the solution was measured at 765 nm by using a spectrophotometer (Shimadzu) and quantified by using a gallic acid standard curve.

### 2.4. Oxygen Radical Absorbance Capacity (ORAC) Assay of Klamin®'s Phenolic Extract

The ORAC assay was performed according to Ninfali et al. [44] and Cao et al. [45], slightly modified. The reaction was carried out using a 96-well plate: 160 *μ*l of 0.04 *μ*M fluorescein in 0.075 M Na-K phosphate buffer (pH 7.0), 20 *μ*l of the diluted phenolic extract, or 20 *μ*l of 100 *μ*M Trolox. The mixture was incubated for 10 min at 37°C in the dark; after this incubation, 20 *μ*l of 40 mM 2,2′-Azobis-(2-methylpropionamidine) dihydrochloride (AAPH) solution was added. The microplate was immediately placed in a microplate reader (Thermo Scientific Fluoroskan Ascent F2 Microplate) and the fluorescence recorded (excitation and emission wavelengths of 485 and 527 nm, respectively) every five minutes for 60 min. The ORAC value refers to the net area under the curve of fluorescein decay in the presence of the Klamin® phenolic extract or Trolox, minus the blank area. The activity of the sample was expressed by *μ*mol of Trolox equivalents (TE)/g of Klamin®, with the following equation:

(1) $$\begin{array}{c} ORAC\left(\mu mol\;TE/g\right)=kah\left[\frac{\left(S_{sample}-S_{blank}\right)}{\left(S_{Trolox}-S_{blank}\right)}\right], \end{array}$$

where *k* is the final dilution of the water soluble extract, *a* is the ratio between the volume (liters) of the water soluble extract and grams of Klamin®, *h* is the final concentration of Trolox expressed as *μ*mol/l, and *S* is the area under the curve of fluorescein in the presence of sample, Trolox, or buffer solution. All the reaction mixtures were prepared in triplicate, and at least three independent assays were performed for each sample.

### 2.5. Cell Cultures and Treatment

Cells were cultured with RPMI 1640 medium (Celbio srl, Milan, Italy) supplemented with 10% fetal bovine serum (Gibco-Invitrogen, Milan, Italy) and 1% antibiotics (50 mg ml^−1^ penicillin and 50 mg ml^−1^ streptomycin). Cells were maintained in a humidified 5% CO_2_ atmosphere at 37 ± 0.1°C. For dose- and time-dependent assay, LAN5 cells were treated with 50, 100, 200, 400, and 800 ng/*μ*l of extract for 3, 24, 48, and 72 hours. For the DCFH-DA, MitoRed, and JC-1 assays and immunofluorescence experiment, the AFA extract was utilized at 800 ng/*μ*l for 24 h. The treated and control cells were analyzed by using microscopy (Axio Scope 2 microscope; Zeiss, Germany). In the experiments in which the A*β* peptide effect was analyzed, 40 *μ*M of a recombinant peptide produced according to Carrotta et al. [46] was utilized.

### 2.6. Determination of Cell Viability

Cell viability was measured by MTS assay (Promega Italia S.r.l., Milan, Italy). MTS was utilized according to the manufacturer's instructions. After cell treatments, the incubation was continued for 3 hours at 37°C in 5% CO_2_. The absorbance was read at 490 nm on the Microplate reader WallacVictor 2 1420 Multilabel Counter (Perkin Elmer Inc., Monza, Italy). Results were expressed as the percentage MTS reduction with reference to the control cells.

### 2.7. Oxidation Kinetics

LAN5 cells were treated with 1 mM TBH or 40 *μ*M A*β* to induce oxidative stress and cotreated with five different concentrations of the AFA extract (50, 100, 200, 400, and 800 ng/*μ*l). The production of reactive oxygen species (ROS) was evaluated using 2′,7′-dichloro-diidrofluorescineacetate (DCFH-DA) (Molecular Probes, Eugene, OR). The oxidation kinetics was evaluated using the GloMax® Discover System (Promega) in a 96-multiwell plate incubated for 2 hours at 37°C at the excitation wavelength of 475 nm and emission wavelength 555 nm. After treatment, cells were analyzed with the microscope Leica Microsystems (Leica, Heidelberg, Germany). Results were compared with untreated cells, used as control.

### 2.8. ROS Generation and Mitochondrial Transmembrane Potential Modification

To assess ROS generation, the cells were incubated as mentioned above. Afterwards, cells were incubated with 1 mM DCFH-DA in PBS for 10 min at room temperature in the dark. After washing with PBS, the cells were analyzed by a fluorescence microscope (Axio Scope 2 microscope; Zeiss, Oberkochen, Germany) and a fluorimeter (Microplate reader WallacVictor 2 1420 Multilabel Counter; PerkinElmer Inc.) for fluorescence intensity. Then, cells were treated with 800 ng/*μ*l of the AFA extract and incubated for 30 min at 37°C with 2 mM JC-1 using the MitoProbe JC-1 assay kit (Molecular Probes, Eugene, OR, USA) fluorescent dye. CCCP (carbonyl cyanide 3-chlorophenylhydrazone) (50 *μ*m), a mitochondrial membrane potential disrupter, was used as positive control. Fluorescence emission shift of JC-1 from red (590 nm) to green (529 nm) was evaluated by fluorimeter (Microplate reader WallacVictor 2 1420 Multilabel Counter; PerkinElmer, Inc.) and fluorescence microscope (Axio Scope 2 microscope; Zeiss) equipped with 488 nm excitation laser. The mitochondrial production of superoxides was analyzed by fluorescence using the MitoSOX Red reagent. After the treatment, the cells were washed with PBS and incubated with 5 *μ*M MitoSOX reagent working solution for 10 min at 37°C in the dark. At the end of the incubation, cells were washed in PBS and analyzed by fluorimeter. MitoSOX fluorescence was measured using supercontinuum white laser (Leica Microsystems CMS, Mannheim, Germany) at the excitation wavelength of 514 nm and recording the emission spectrum in the range 540–640 nm.

### 2.9. Immunofluorescence

10^6^/ml LAN5 cells were cultured on Lab-Tek II Chambered Coverglass (Nunc) and treated as described above. After washing in PBS, the cells were fixed in 4% paraformaldehyde for 30 min and stored at 4°C. After incubation with 3% BSA/PBS for 1 h, the cells were immunostained with anti-phosphorylated-NF*κ*B (1 : 100; Cell Signaling) antibody at 4°C overnight. After washing in PBS, the samples were incubated with anti-rabbit Cy3-conjugate secondary antibody (1 : 500; Sigma). For nuclear staining, the cells were incubated with Hoechst 33258 (5 *μ*g/ml) for 20 minutes. After washing, the cells were visualized by using a Leica DM5000 upright microscope (Leica Microsystems, Heidelberg, Germany) at 20x magnification.

### 2.10. ELISA

Supernatant of LAN5 cultured cells was centrifuged at 14,000 rpm for 30 min at 4°C. 100 *μ*l of the supernatant were used to measure interleukin-1*β* and interleukin-6 (Cloud-Clone Corp) according to manufacturer's instructions. The samples were red on iMark™ Microplate Absorbance Reader at 450 nm.

### 2.11. Kinetics of A*β* Aggregation

Samples containing A*β* (40 *μ*M) alone and A*β* with AFA extract (A*β*-AFA) (800 ng/*μ*l) were placed in a 96 black multiwell plate at which thioflavin T (8 *μ*M) was added. The kinetics was followed by using a fluorimeter at the wavelength of 485 nm at 37°C for 8 hours until the samples arrived to the plateau. The formation and mean size of A*β* aggregates was evaluated by using a fluorescence microscope (Leica Microsystems, Heidelberg, Germany).

### 2.12. Statistical Analysis

All experiments were repeated at least three times and each experiment was performed in triplicate. The results are presented as mean + SD. A one-way ANOVA was performed, followed by Bonferroni post hoc test for analysis of significance. Results with a *p* value < 0.05 were considered statistically significant.

## 3. Results

### 3.1. Characterization of the Water-Soluble Klamin® Extract

**Figure 1:**
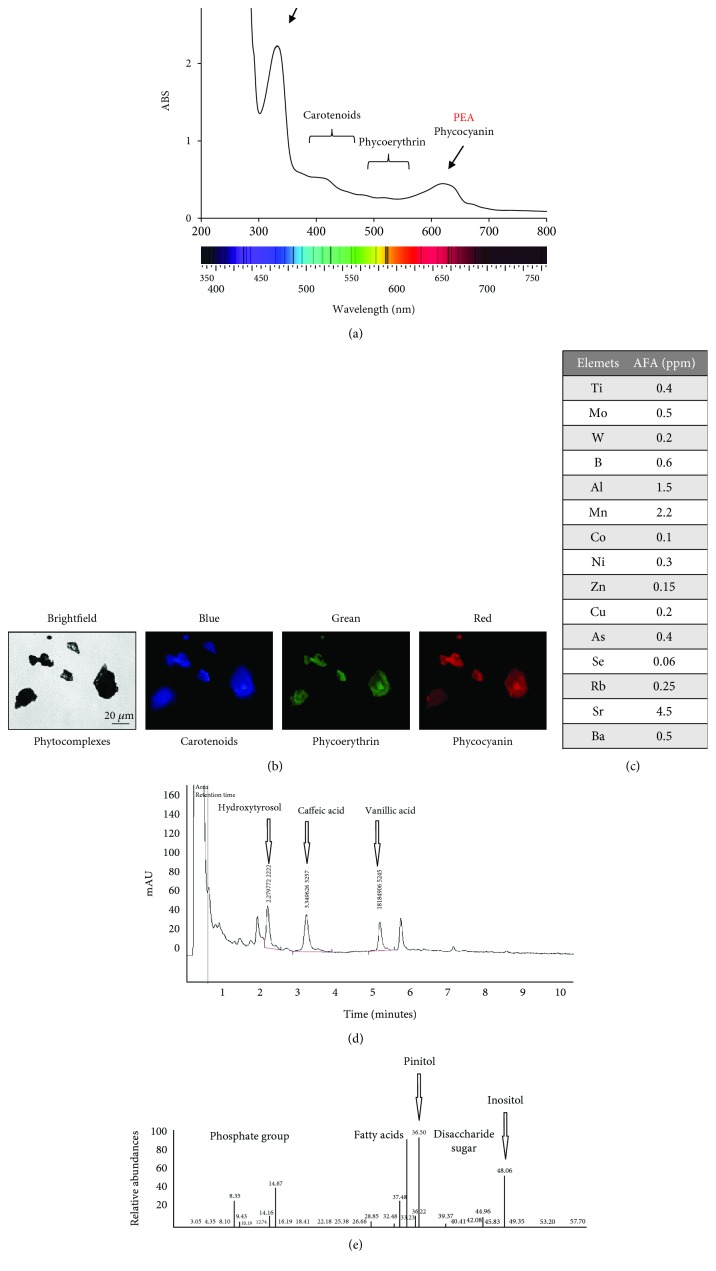
Hydrosoluble Klamin® extract compounds. (a) Spectrophotometric measure of the extract presenting different peaks of absorption both in the spectrum range between 310 and 360 nm (MAAs) and in the spectrum range of 400–600 nm. (b) Fluorescence analysis of phyco-complexes. (c) Spectrum of minerals obtained by ICP-MS analysis. (d) Phenolic components separated by HPLC. (e) Spectrum of carbohydrate obtained by GC-mass analysis.

With the aim to test the biological effect of AFA compounds on *in vitro* cell culture, the extract was dissolved in PBS and submitted to different chemical analyses. Spectrophotometer absorption measures in the range between 310 and 360 nm indicated the presence of mycosporine-like amino acids (MAAs), whereas absorption in the spectrum range between 400 and 600 nm indicated the presence of carotenoids, phycoerythrins, and phycocyanins (Figure 1(a)). The presence of phyco-complexes was confirmed by using fluorescence microscope at different wavelengths, by using appropriate excitation and emission filters (Figure 1(b)).

By ICP-MS, a spectrum of minerals was identified and an unusual presence of molybdenum and tungsten was revealed (Figure 1(d)). The polyphenolic component was separated by HPLC, and peaks corresponding to hydroxytyrosol, vanillic acid, and caffeic acid were detected (Figure 1(d)). Presence of inositol and pinitol was revealed by GC-Mass spectrum (Figure 1(e)). Thus, the hydrosoluble Klamin® extract contains molecules with potential bioactive activity.

AFA extract antioxidant activity expressed as *μ*mol of Trolox equivalents (TE) per gr of extract for ORAC assays and as mg of gallic acid equivalents per gr of extract for the Folin-Ciocalteu (F-C) assay are reported in Table 1.

### 3.2. Klamin® Is Not Cytotoxic

**Figure 2:**
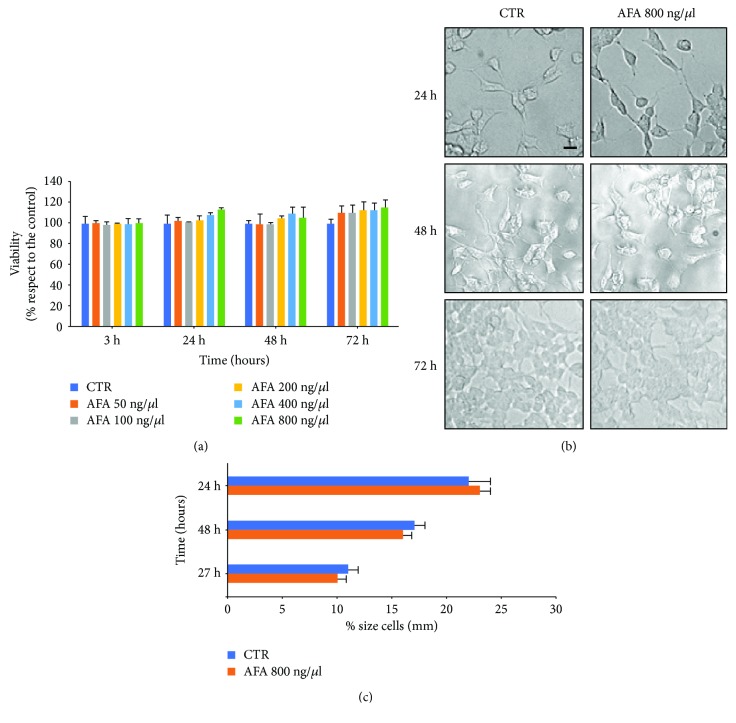
Cytotoxicity of the AFA extract on LAN5 cells. (a) MTS cell viability assay. (b) Representative morphological images of untreated cells (CTR) and after 24, 48, and 72 hours from the addition of 800 ng/*μ*l of the extract. (c) Analysis of the body size of untreated cells (CTR) and cells treated with the AFA extract at different concentrations and times. Bar: 50 *μ*m.

To evaluate the possible toxicity of the AFA extract, different concentrations were added to LAN5 cells, and after incubation at different times, an MTS assay was performed.  Figure 2(a) shows that no toxicity was detected at all concentrations and times compared with the control. The result was confirmed by the morphological observation of the cells treated with the highest AFA extract concentration and incubated at different times (Figure 2(b)). Correct cell shape was observed and measures of the cell body confirmed the absence of any cell damage (Figure 2(c)).

### 3.3. Klamin® Prevents Oxidative Stress

**Figure 3:**
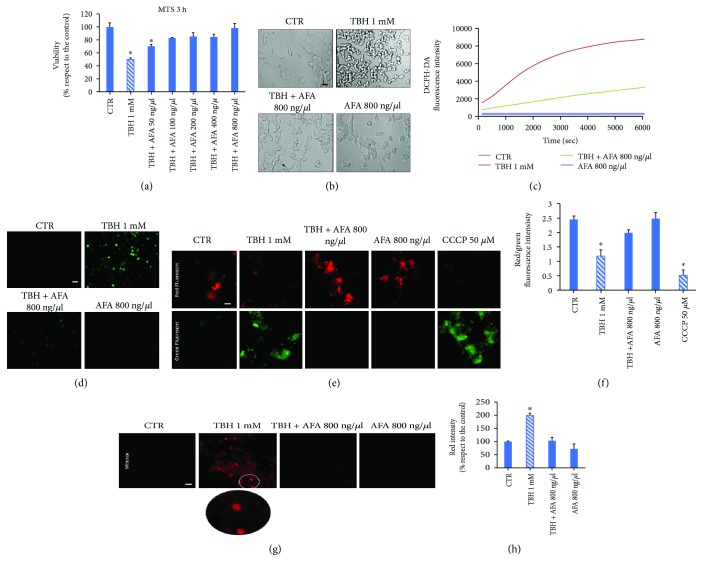
AFA extract protects LAN5 cells from oxidative insult. (a) Viability assay on untreated cells (CTR) or cells treated with TBH or cotreated with TBH and AFA extracts at increasing concentrations for 3 hours. (b) Representative morphological images of untreated cells (CTR) or cells treated with TBH or cotreated with TBH and the AFA extract and treated with the AFA extract. (c) Oxidation kinetics of LAN5 cells alone (CTR) or in the presence of TBH or TBH and AFA at two concentrations measured by the DCFH-DA assay. (d) Fluorescence microscopy images of untreated cells (CTR) and cells treated with TBH or cotreated with TBH and the AFA extract. Bar: 50 *μ*m. AFA extract protects against mitochondrial damage. (e) Fluorescence microscope images of cells untreated (CTR) or treated with TBH alone or with the AFA extract or with CCCP and submitted to JC-1. (f) Values of the ratio between the red and green fluorescence intensities as compared with the control. (g) MitoSOX assay on control cells (CTR) and cells treated with TBH, with TBH and the AFA extract, with AFA alone, and with CCCP. (h) Histogram representing the red fluorescence intensity. Bar: 50 *μ*m.

The AFA extract protective property was evaluated by treating LAN5 cells with TBH alone or in combination with increasing concentrations of the AFA extract and after 3 hours of incubation. As detected by MTS assay, the AFA extract is able to inhibit TBH-induced toxicity at 50 ng/*μ*l in a dose-dependent manner (Figure 3(a)); the results were also confirmed by microscopic observation in which a significant recovery of the altered cell morphology was observed (Figure 3(b)). Furthermore, Klamin® antioxidant ability was analyzed by using DCFH-DA assay. Presence of Klamin® decreases TBH-induced ROS generation at 800 ng/*μ*l (Figure 3(c)). The result was also confirmed by fluorescence microscope inspection (Figure 3(d)), in which untreated, or AFA extract treated cells, did not show any fluorescence; in contrast, cells treated with TBH showed green fluorescence due to ROS generation. The result suggests that the components of AFA extract, such as carotenoids, phycoerythrins, phycocyanins, MAAs, and polyphenols, play a significant role as antioxidant agents.

The possible protective effect of the AFA extract on mitochondrial dysfunction was also evaluated. LAN5 cells treated with TBH and CCCP, as positive control, show an intense green fluorescence indicating that a high depolarization of the mitochondrial membrane is occurred. Cells cotreated with TBH and AFA extract, or alone with AFA extract, showed instead a higher red fluorescence emission similar to the untreated control (Figure 3(e)). The red/green fluorescence ratio is represented in the histogram in Figure 3(f).

The ability of the AFA extract in counteracting the mitochondrial superoxide ions, generated as byproducts of oxidative phosphorylation, was investigated through MitoSOX assay. LAN5 cells treated with TBH exhibited a high fluorescence intensity. On the contrary, the cells treated with the AFA extract alone, or with TBH and Klamin®, do not show any fluorescent signal (Figure 3(g)). The fluorescence was quantified by fluorimeter analysis and illustrated in the relative histogram (Figure 3(h)). This data is in agreement with the presence of biomolecules such as hydroxytyrosol, vanillic acid, and caffeic acid in the AFA extract, whose antioxidant properties are well known, plus carotenoids and the specific cyanobacterial antioxidant molecules such as phycocyanins and phycoerythrins.

### 3.4. Klamin® Protects LAN5 Cells from Toxicity and Oxidative Stress Induced by A*β*

**Figure 4:**
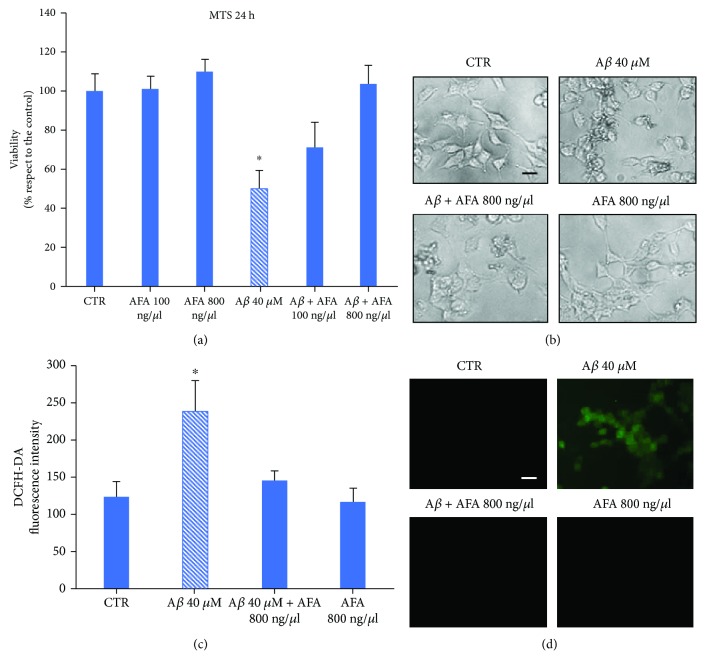
AFA protects against A*β*-induced toxicity. (a) MTS of untreated LAN5 cells (CTR) or cells treated with the AFA extract at two concentrations, with A*β* alone, or with A*β*-AFA at two concentrations for 24 hours. (b) Morphological representative images of samples indicated in (a). (c) DCFH-DA assay of untreated LAN5 cells (CTR) or cells treated with A*β* alone or with A*β*-AFA at two concentrations. (d) Fluorescence representative images of samples indicated in (c). Bar: 50 *μ*m.

The potential neuroprotective effect of the AFA extract was evaluated by treating LAN5 cells with A*β* peptide alone and with two AFA extract concentrations and submitted to MTS assay. The A*β*-induced toxicity was inhibited by the coadministration of the AFA extract between 100 ng/*μ*l and 800 ng/*μ*l (Figure 4(a)). Observation of cell morphology confirmed the viability assay results (Figure 4(b)). The antioxidant capacity of AFA extract against the A*β* peptide-induced oxidative stress was evaluated by DCFH-DA assay. Fluorescence analysis indicated that cells treated with A*β* alone exhibit high levels of ROS generation, whereas cotreatment of A*β* and AFA extract (A*β*-AFA) did not produce any fluorescent signal (Figure 4(d)). The same samples were observed at fluorescent microscopy (Figure 4(d)).

### 3.5. Neuroinflammation

**Figure 5:**
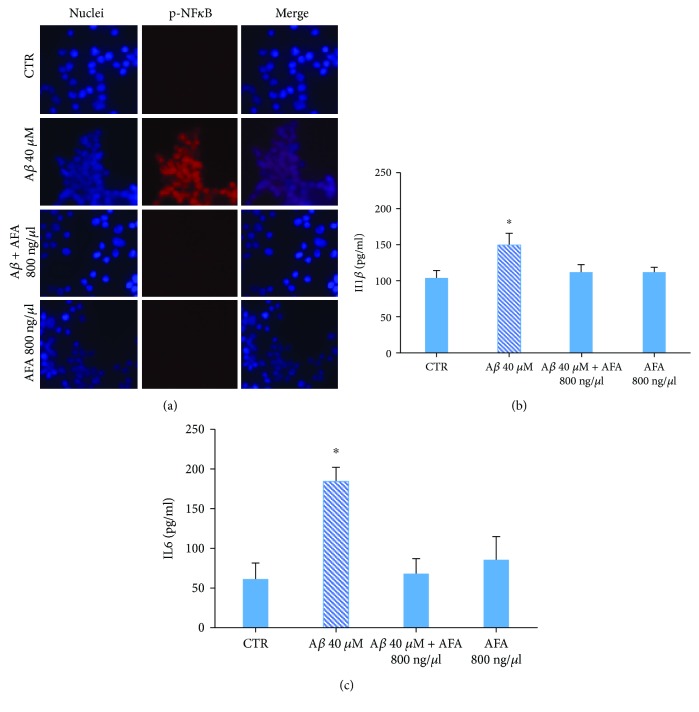
AFA extract inhibits A*β*-induced NF*κ*B nuclear translocation. (a) Immunofluorescence of untreated LAN5 cells (CTR) or cells treated with A*β*, with A*β*-AFA, or AFA alone and incubated with an antibody against p-NF*κ*B. (b–c) Measurement of the level of expression of IL-1*β* and IL-6 in untreated LAN5 cells (CTR) or cells treated with A*β*, with A*β*-AFA, or AFA alone, by the ELISA test.

NF*κ*B is a transcription factor that under stimulation is phosphorylated and translocates from the cytoplasm to the nucleus where several genes including those triggering inflammation responses are activated. We assessed the ability of AFA to inhibit A*β*-induced inflammation by analyzing activated NF*κ*B (p-NF*κ*B) localization. Immunofluorescence analysis showed that after LAN5 A*β* stimulation, p-NF*κ*B was localized in the nucleus, whereas after AFA or A*β*-AFA treatment, no presence of p-NF*κ*B in the nuclei of the cells (Figure 5(a)) was detected. In addition, to take another evidence that Klamin® protects the cells against A*β* inflammatory response activation, the expression of cytokines1*β* and 6 (IL1*β* and IL6) was measured. The data shown in Figures 5(b) and 5(c) indicate that, in agreement with NF*κ*B activation, the AFA extract significantly reduces the expression of proinflammatory cytokines.

### 3.6. Klamin® Affects A*β* Peptide Aggregation

**Figure 6:**
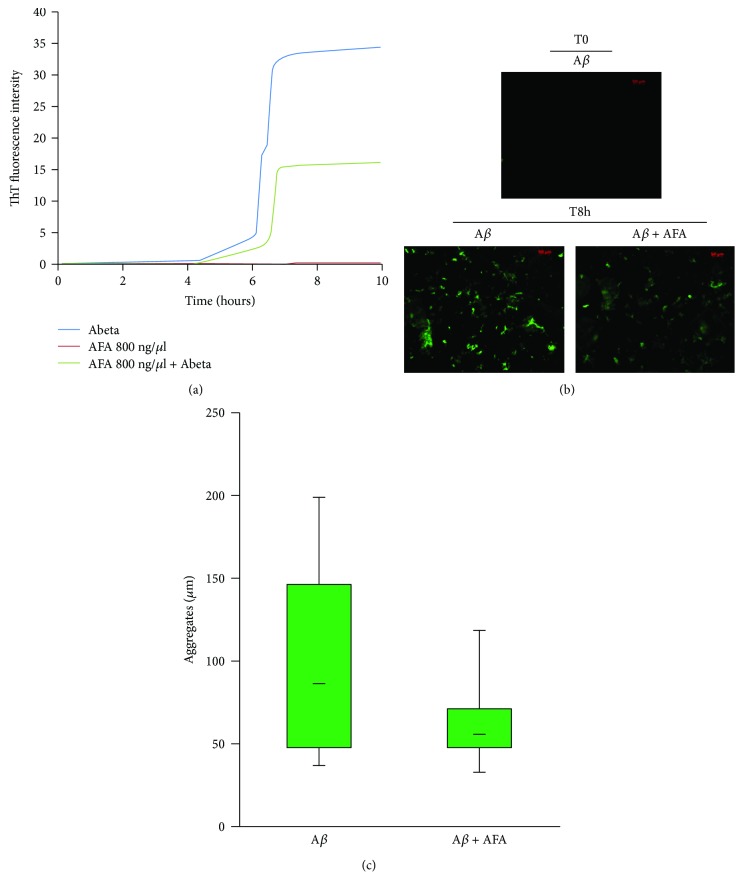
Effects of the AFA extract on A*β* aggregation. (a) Kinetics of A*β* aggregation alone or with AFA incubated with ThT. (b) Fluorescence microscopic images representing size and morphology of A*β* aggregates at *t* = 0 and *t* = 8 in the absence or presence of AFA. (c) Histogram of medium size of A*β* aggregates formed in the absence or presence of AFA at *t* = 8.

The effect of the AFA extract on the aggregation kinetics of A*β* peptide was measured in presence of thioflavin T (ThT). Results, showed in Figure 6, indicated that the AFA extract interferes with the A*β* aggregation kinetics. Fluorescence microscopy analysis on samples containing A*β* alone, or A*β* plus AFA extract, was done at time O (*t* = 0 and after 8 hours (*t* = 8). At *t* = 0, no signal was detected in any samples (Figure 6(a)). At *t* = 8 in A*β* sample, a diffuse fluorescence is detectable and objects with an average size of about 90 *μ*m were present (Figure 6(b)). On the contrary, a slight fluorescence was visible in A*β* + AFA extract sample in which objects having mean size of 50 *μ*m was present (Figure 6(c)). These results reveal that the AFA extract affects A*β* aggregation inducing formation of smaller aggregates.

**Figure 7:**
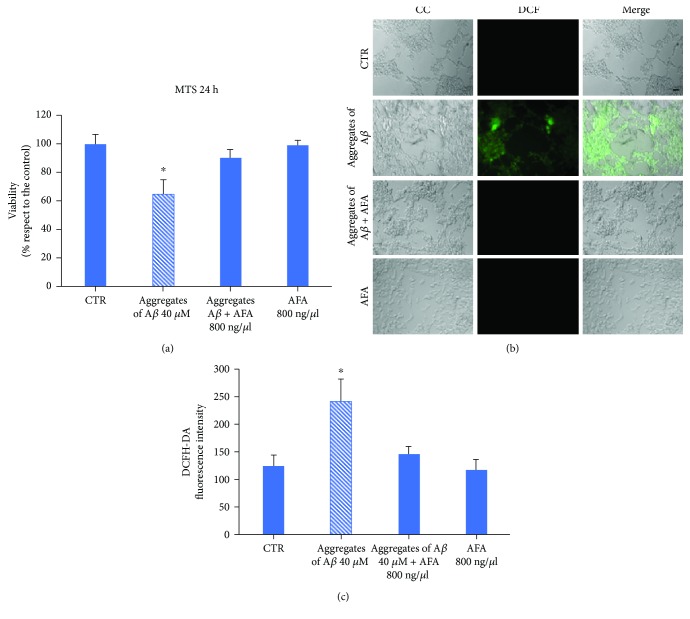
A*β*-AFA aggregates do not affect toxicity and oxidative stress. (a) MTS assay of untreated LAN5 cells (CTR) or cells treated with A*βag*, A*β*-AFA*ag*, or AFA alone for 24 hours. (b) DCFH-DA assay of untreated LAN5 cells (CTR) or cells treated with A*βag*, A*β*-AFA*ag*, or AFA alone. (c) Fluorescence intensity of the samples shown in (b). Barr: 20 *μ*m.

Furthermore, the toxicity and antioxidant ability of the A*β* aggregates formed in the absence or in the presence of the AFA extracts on LAN5 cells was evaluated through MTS (Figure 7(a)) and DCFH-DA assay (Figure 7(b)). Aggregates of A*β* peptide (A*βag*) caused a reduction of about 40% of cell viability when compared with the control. On the contrary, the addition of A*β* + Klamin® aggregates (A*β*-AFA*ag*) did not significantly affect cell viability. Microscope fluorescence images performed after DCFH-DA assay showed that a diffuse green fluorescent signal was mainly present in cells treated with A*βag* while no signal was revealed after treatments with A*β* + AFA*ag*. Furthermore, no significant morphological differences were observed from cells incubated with A*β* + AFA*ag* or control cells or cells treated with the AFA extract (Figure 7(b)).

## 4. Discussion

Cyanobacteria are a source of structurally different bioactive compounds with potential nutraceutical, pharmaceutical, and cosmeceutical employment [47]. The AFA extract Klamin® contains secondary metabolites such as MMAs, polyphenols, sugars, and different minerals. By spectrophotometer analysis, MMAs were detected in the UV absorbing range of 310–360 nm and carotenoids, phycoerythrins, and phycocyanins in the visible spectrum of light between 400 and 650 nm. MMAs are synthesized and accumulated by the algae as defense against exposition to environmental UV [48]. Use of high-resolution nuclear magnetic resonance (NMR) has evidenced in Klamin® a high concentration of MAAs and in particular of porphyra-334 (P334) and shinorine (Shi), two monoamine oxidase (MAO) inhibitors [49]. These molecules can cross the blood-brain barrier (BBB) and express their MAO-B inhibitory potential in the brain [20]. Most of the mycosporines exhibit high antioxidant activity by scavenging large amounts of reactive oxygen such as superoxide anions and hydroxyl radicals [50]. Thus, their antioxidant properties, born to contrast environmental stress, can be exploited to enhance human health. Molybdenum and tungsten, two transition metals, also known as enzymes cofactors [51, 52] were found in high concentration in Klamin® and, even though additional studies would be required, we can plausibly expect that they can activate biochemical pathways with a beneficial effect. Presence of polyphenols such as hydroxytyrosol, vanillic acid, caffeic acid; of phytosynthetic pigments such as carotenoids and phycoerythrins, endowed with well-known antioxidant properties: and most of all of the AFA-phycocyanins, which have shown to be the most powerful antioxidants among all purified molecules [14], suggests that the AFA extract can be regarded as a valuable resource for human nutrition and health.

Klamin® does not show any toxicological risks on the LAN5 cell line even at high doses, indicating that it does not contain any unhealthy by-products for this test. The radical scavenging activity of the AFA extract was confirmed by *in vitro* experiments in which an oxidant agent was used. The antioxidant activity found with the AFA extract is probably particularly strong thanks to the synergic effect of all its various components. TBH-induced mitochondrial dysfunction was also prevented by the administration of Klamin®, indicating that its scavenging effect is also extended to mitochondrial ROS. TBH-induced mitochondrial membrane damage is inhibited by Klamin®, indicating that it exerts a protective role in the cell's fundamental organelle, involved in several pathological dysfunctions.

Free radical generation is one of the main causes of aging and aging-related diseases in humans, and antioxidants may have a positive antiaging effect [53]. The use of natural nutritional supplement could be an important milestone for the prevention and treatment of neurodegenerative diseases. Here, we found, for the first time, that the AFA extract Klamin® has a beneficial effect on neurons in which toxicity was induced by A*β* peptide, suggesting that the AFA extract, consumed as a nutraceutical, could play a significant neuroprotective role. Furthermore, the AFA extract exerts anti-inflammatory activity as a response to A*β* toxic stimulus. Inflammation is a pathological mechanism underlying many chronical diseases, including neurodegenerative diseases, and chronic inflammation have been found in the brain of early AD [54]. The anti-inflammatory function of the molecular components of Klamin® is mediated by its inhibition of the nuclear factor kappa B (NF*κ*B) activation and by its decreasing the production of proinflammatory cytokines such as IL-6 and IL-1*β*. However, we cannot exclude that Klamin® neuroprotective activity can be exerted through other components. By molecular docking, simulation studies have been demonstrated that phlorotannins derived from Eisenia bicyclis, a Japanese alga, can be a potential inhibitor of *β*-amyloid cleavage enzyme (BACE1) activity, a protease involved in A*β* formation [55].

Fibrillogenesis of A*β* is a relevant event, leading to deposition of amyloid plaques in AD brain. Use of inhibitors can prevent this process [56]. Klamin® interferes on A*β* aggregation stabilizing A*β* aggregates in a protective way to reduce oxidative stress. The kinetics of amyloid formation can be described in a sigmoid curve subdivided in three stages: (a) the slow lag nucleation phase, (b) the fast-exponential elongation phase, and (c) the saturation phase. Klamin® blocks the elongation phase, producing aggregates of shorter dimensions. We can suppose that the various antioxidant components of AFA extract, from its polyphenols to its phycocyanins, interact with A*β*, inhibiting its extension and destabilizing the preformed fibrils. It is known that antioxidants, containing one or more phenolic rings, are able to interact with the aromatic residues of the amyloid peptides, destabilizing their well-ordered self-assembly process [37]. Thus, Klamin® could have a disaggregating activity on A*β*.

In conclusion, Klamin® is a reservoir of effective molecules with numerous health benefits. The intrinsic antioxidant, anti-inflammatory, and antifibrillogenesis properties of its compounds suggest that it could be used as an innovative approach for the treatment and/or prevention of neurodegenerative disease.

## Figures and Tables

**Table 1:** Antioxidant capacity of the hydrosoluble Klamin® extract assayed by ORAC and F–C reducing capacity.

Test	Value
ORAC	86.54 ± 15.6 *μ*mol TE/g
FOLIN	86 ± 11.4 mg/l

## Data Availability

The data used to support the findings of this study are available from the corresponding author upon request.
